# Eating Behaviours in Sportswomen from the Silesian Training in Different Sports Disciplines

**DOI:** 10.3390/ijerph192416843

**Published:** 2022-12-15

**Authors:** Magdalena Rutkowska, Mariola Czajkowska, Iwona Nowakowska, Anna Kowalczyk, Tomasz Król, Magdalena Dąbrowska-Galas, Violetta Skrzypulec-Plinta

**Affiliations:** 1 Department of Kinesitherapy and Special Methods, Department of Physiotherapy, Faculty of Health Sciences in Katowice, Medical University of Silesia in Katowice, Medyków 12 Street, 40-752 Katowice, Poland; 2 Department of Propaedeutics of Obstetrics, Department of Women’s Health, Faculty of Health Sciences in Katowice, Medical University of Silesia in Katowice, Medyków 12 Street, 40-752 Katowice, Poland; 3 Department of Balneoclimatology and Biological Regeneration, Department of Physiotherapy, Faculty of Health Sciences in Katowice, Medical University of Silesia in Katowice, Medyków 12 Street, 40-752 Katowice, Poland; 4 Department of Physiotherapy, Faculty of Health Sciences in Katowice, Medical University of Silesia in Katowice, Medyków 12 Street, 40-752 Katowice, Poland; 5 Department of Reproductive Health and Sexuology, Department of Women’s Health, Faculty of Health Sciences in Katowice, Medical University of Silesia in Katowice, Medyków 12 Street, 40-752 Katowice, Poland

**Keywords:** women, diet, training, sport, physical activity, FAT

## Abstract

Eating disorders are characterized by abnormal, unhealthy eating habits, and disordered body image. In severe cases, it can cause serious health consequences, including cardiac problems, osteoporosis, infertility, or missing menstrual periods. In women competitively training sports, the main cause for disordered eating behaviours are factors associated with dissatisfaction with their appearance and body image and a need to reduce body weight. Factors related to dissatisfaction with one’s own appearance and body image, a need to reduce body weight, a negative perception of themselves and their bodies, the pressure in sports circles, and stress are predictors for eating disorders. The aim of the study was to compare eating behaviours, body satisfaction, and taking various actions related to body mass reduction among women training competitively in volleyball, athletics, gymnastics, and young women not participating in sports. Material and methods: The study covered a group of girls and young women from the Silesian, Poland, who represented three sports disciplines (volleyball, athletics, and gymnastics), of which 30 girls were used as a control group (B). The study was conducted using a study questionnaire. Results: The study participants ate regularly and consumed all food groups during a week. The majority of girls from A1 (83.33%), A3 (53.33%), and B (80%) groups expressed their dissatisfaction with their body weight. In the group of gymnasts, a positive correlation was noted between the need to reduce body weight and regular eating (r = 0.449; *p* = 0.013). In the group of volleyball players, it was demonstrated that the higher the competitive experience and the greater the training load, the more regular their eating was (r = 0.475; *p* = 0.009). Conclusions: The importance of a diagnosis of early signs of eating disorders in girls and women as a basic component contributing to FAT development implies that further studies in this area, as well as education of the entire sports circle are necessary.

## 1. Introduction

The principles of correct eating are decisive for health and well-being. A well-balanced diet provides nutrients of required quality and quantity, which are necessary for normal body functioning, optimum health, and good mood when supplied on a regular basis, and in required proportions. A demand for nutrients and nutritional requirements depends on age, gender, performed work, physiological status, and physical activity. [1,2,3].

Eating disorders are characterized by abnormal, unhealthy eating habits and disordered body image. In severe cases, it can cause serious health consequences, including cardiac problems, osteoporosis, infertility, or missing menstrual periods [4,5]. It is well known that disordered eating behaviours or eating disorders are more common among elite and competitive athletes compared to the normal population, especially in disciplines that emphasize thinness, low weight, or lean aesthetics [6,7]. Although male athletes can be affected by eating disorders, females represent about 90% of those who need medical care [8,9].

According to recent reports in women competitively training, the main cause for disordered eating behaviours are factors associated with dissatisfaction with their appearance and body image, a need to reduce body weight [10,11,12,13,14,15,16,17], a negative image of themselves and their body [10,14], the pressure among sports circles [10,15,16,18], and the pressure associated with reduction in body weight and with body shape [13,16,19].

The majority of studies show the prevalence of eating disorders in female athletes ranging from 18–42%, compared to 2–12% in non-athletes [20,21]. The rate of eating disorder varies by sport and concerns about 25–42% female in aesthetic sports, 24% in endurance sports, 17% in technical sports, and about 16% in ballgame sports [15,20,21]. Results of other studies showed that disordered eating behaviours concerns about 70% of athletes, mostly in lean sports, regardless of level of competition [13,22] 

Disordered eating behaviours leading to subclinical and clinical eating disorders are the first factor that may result in serious consequences to health, such as the multicomponent disorder called the Female Athlete Triad (FAT), consisting of disordered eating, menstrual dysfunction, and low bone mineral density [23,24,25,26,27].

Results of studies on eating conducted on girls and women competitively training in various sports disciplines are not unambiguous, mainly due to different tools used for analyses. As a consequence, screening studies on eating disorders are limited. Currently, no “gold standard” tool is available, and authors of reports concerning early identification of eating disorders provide a set of screening questions that should be used for a sportsperson’s evaluation [24,25,27]. In Poland, the research focused on negative consequences of physical efforts in relation to menstrual cycle dysfunctions in girls and women in competitive training sports. However, no reports are available on disordered eating behaviours and/or eating disorders [28,29,30,31,32]. 

Young female athletes are still in a process of emotional, mental, and physical development. The challenge is to know the prevalence of disordered eating behaviuors to promote healthy well-being by trainers and physicians. The lack of a clear research hypothesis is due to the fact that these are preliminary studies with a large number of variables. This research focused on exploratory research questions; thus the aim of the study was to compare eating behaviours, body satisfaction, and taking various actions related to body mass reduction among women training competitively in volleyball, athletics, and gymnastics, and young women not participating in sports. 

## 2. Materials and Methods

### 2.1. Sample and Setting

The study was conducted in 2020–2021 and covered a group of 120 girls and young women aged 16 to 19 years old, living in the Silesia. Group A—a study group (*n* = 90) consisted of women training competitively in volleyball (group A1, *n* = 30), athletics (group A2, *n* = 30), and gymnastics (group A3, *n* = 30). Girls and young women were associated in sports clubs and/or attended a sports school. The athletes represented their clubs at national and international competitions. Group B—a control group (*n* = 30) consisted of women and girls who have not and do not train in competitive sports. Their physical activity was limited to school classes or amateur fitness classes. The participants from the study group were selected by non-probability sampling. The girls were recruited at sports clubs at which they were associated. The participants in group B were selected randomly from girls and young women aged 16 to 19 years from the same area of residence.

The inclusion criteria for the study group were age (16–19 years old), and actively competing in sports at the national and international level. The exclusion criteria for the athletes group were; no participation in training, resulting from a sports injury; eating-related diseases, diagnosed currently or in the past; no consent to participate in the study; and depressive disorders. In the control group, the inclusion criteria was age (16–19 years), while exclusion criteria were; eating-related diseases, diagnosed currently or in the past; depressive disorders; and no consent to participate in the study. Those respondents who did not fully complete the questionnaire were also removed from the study.

The questionnaire was delivered to the participants in person; to the athletes, during trainings (following arrangements with their trainer), and to the control group, during classes at school (following arrangements with a school director and their teacher). In the athletes group, the study was conducted during the training cycle after a one-month holiday regeneration period and preparatory sports camps initiating intense training cycles. Each respondent was informed that the study was confidential. They were also informed about the study project aims and assumptions, and study methods. The participation in the study was voluntary, and the study was performed after receiving a written consent to participate in the study from respondents. In the case of minors, consent by their legal guardians was obtained, in accordance with the Declaration of Helsinki.

The study received a positive opinion of the Ethics Committee at SUM in Katowice—decision No. PCN/0022/KB/166/20.

### 2.2. Outcome Measures

A pilot study was conducted before the actual survey study. It was designed to verify whether the questionnaire structure was substantially correct, and the technique of asking questions was right. The pilot study was conducted in 10 girls training competitively and 10 girls not training in sports. The results obtained in the pilot study were not included in the main study.

The questionnaire consisted of 2 parts: The first part concerned social and demographic data, a family interview, a current health status, lifestyle and diseases, and physical activity (trained sports discipline, competitive experience, duration of an individual training, number of training sessions per week, total training time per week, as well as injuries resulting from physical training). The questions concerning eating, in the second part of the questionnaire, were formed on the basis of methodology described in other publications on this subject, and questionnaires developed by societies dealing with FAT [15,17,24,27,33].

Questions concerning eating covered the last seven days. The respondents were asked about the regularity of their meals, the number of meals eaten during the day, and the frequency of eating specific products during the week, as well as medicines, vitamins, and dietary supplements taken. This part also included questions on satisfaction with body weight, the need to increase or reduce body weight, and methods of body weight control.

### 2.3. Statistical Analysis

The study results were entered into a spreadsheet, and then analysed for statistical relationships (IBM SPSS Statistics 23, Armonk, NY, USA).

The analysed parameters were presented using descriptive statistics (M, SD, Me, 95%CI), and the normality of quantitative variable distribution was verified with the Kolmogorov–Smirnov test. The independent variables in each group were compared using the one-way analysis of variance (ANOVA), and post-hoc tests. The effect size was measured using Cohen’s d (values of up to 0.06 indicate a small effect, values ranging from 0.06 to 0.14 correspond to a medium effect, and above 0.14, indicate a large effect). The relationship between qualitative parameters was evaluated by the χ^2^ compliance test. When assumptions of that test were not met, Fisher’s exact test was employed. Cramér’s V was used to determine the strength of the association or the effect in these tests (values up to 0.3 indicate the low strength, 0.3 to 0.5 correspond to the medium strength, and above 0.5 indicate a high strength of the effect). Relationships between quantitative variables were evaluated by an analysis of correlations using Pearson’s r. The standard threshold of α = 0.05 was considered as a statistically significant level [34].

## 3. Results

The general characteristics of studied respondents (age, body weight, body height, and BMI) are provided in Table 1. Table 1 also shows the differences in described parameters between individual groups.

Characteristics of sports activities in terms of competitive experience, duration of individual training, and the total training time per week for the study group are shown in Table 2. The post-hoc tests showed that group A3 (gymnasts) had the highest competitive experience (M = 8.03; SD =2.220) versus group A2 (athletes) (M = 6.20; SD = 2.074), *p* = 0.005, Cohen’s d = 0.85; 95%CI [0.47; 3.20] and group A1 (volleyball players) M = 6.03; SD = 1.451), *p* < 0.001, Cohen’s d = 1.09; 95%CI [0.80; 3.20]. In addition, the longest training sessions were observed in group A3 (M = 2.60; SD =0.814) versus group A2 (M = 2.07; SD = 0.450), *p* = 0.009, Cohen’s d = 0.90; 95%CI [0.11; 0.95] and group A1 (volleyball players) M = 6.03; SD = 1.451), *p* = 0.001, Cohen’s d = 1.47; 95%CI [0.22; 0.98].

**Figure 1:**
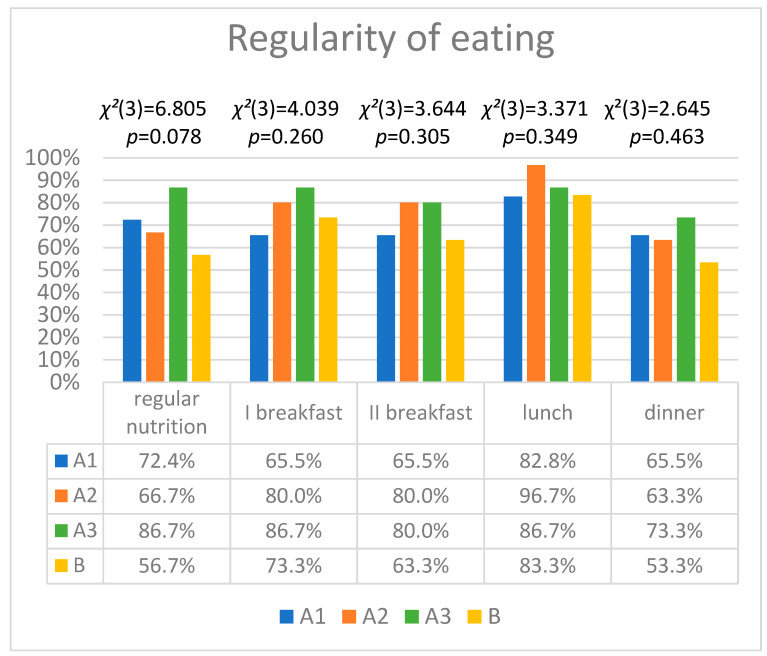
The regularity of eating and of having meals regularly for girls from the sports groups (A1, A2, A3) and from the control group (B). χ^2^—test result; degrees of freedom—df provided in brackets; *p*—statistical significance.

The regularity of eating are shown in Figure 1. No statistical significance was observed between the groups for analysed variables.

The study subjects ate products from all food groups (dairy, fish, meat and cold meats, farinaceous products, pastas, grouts, rice, vegetables, and fruit) during the week. Litres of water drank on average by girls from groups A1, A2, and A3 drank were 2.43 (SD = 0.40), 2.48 (SD = 0.72), and 2.45 (SD = 0.35) litres of water a day, respectively, while the girls from group B drank 1.98 (SD = 0.59) litres of water a day. They also informed that they had sweet and salty snacks during the week. Statistical differences were found for the following areas: frequency of eating fish F (3;85.97) = 2.82; *p* = 0.046; η² = 0.06, farinaceous products F (3;100.73) = 2.70; *p* = 0.049; η² = 0.06, and of fluid intake, which proved to have a large size effect, explaining 15% of the total variability in results observed for the measured test assessment, F (3;89.37) = 6.81; *p* < 0.001; η² = 0.15. No statistically significant differences were observed for other parameters (Table 3).

Post-hoc tests were performed to determine groups in which differences concerning fluids and water uptake occurred. When compared to control group B (M = 1.92; SD = 0.657), girls from group A2 had the highest result for this parameter (M = 2.48; SD = 0.737), *p* = 0.016, Cohen’s d = 0.80; 95%CI [−1.06; −0.08], followed by girls from group A3 (M = 2.45; SD = 0.356), *p* = 0.002, Cohen’s d = 1.05; 95%CI [−0.91; −0.16], and group A1 (M = 2.43; SD = 0.417), *p* = 0.004, Cohen’s d = 0.95; 95%CI [−0.90; −0.12]. Intergruop relationships regarding the consumption of fish and flour products are presented in Table 3.

The use of vitamins and dietary supplements in the diet represented another analysed parameter. A statistically significant difference between the studied groups was found in the area of vitamin intake (*p* = 0.005). Cramér’s V (V = 0.38) indicates a medium size effect (Table 4). The respondents most frequently took pharmaceutical products containing vitamins C and D, while supplements used by the studied girls were mainly formulations containing magnesium and cod liver oil.

In all studied groups, the percentage of girls smoking cigarettes was very low, while alcohol consumption in the group of girls training actively was lower than in the control group, χ^2^(6) = 23.28; *p* = 0.001. The effect size expressed using Cramér’s V was medium (V = 0.31), (Table 5).

The majority of girls from groups A1 (83.33%), A3 (53.33%) and B (80%), expressed their dissatisfaction with their body weight. The largest number of respondents declaring the need to reduce their body weight was noted in group A3 (56.70%). A total of 76.67% and 70% of girls from groups A1 and A2, respectively, did not report the need to reduce their body weight. A total of 53.33% girls from groups A1 and B applied some methods to control their body weight. Statistical significance was observed for all areas presented in Table 6.

Correlations between eating habits (regularity of eating and regular taking of specific meals) and satisfaction with body weight, and the need to reduce it, related to the individual sports disciplines trained in by sportswomen, and their use of methods for body weight control, showed two statistically significant relationships. In the group of gymnasts, a positive relationship was noted between the need to reduce body weight and regular eating (r = 0.449; *p* = 0.013). In the group of volleyball players, a relationship between the need to reduce body weight and regular eating of supper gave a statistically significant negative result (r = −0.409; *p* = 0.027), (Table 7).

In the sportswomen groups, correlations were noted between regular eating, satisfaction with body weight, the need to reduce it, and the use of methods for body weight control, versus competitive experience, the number of training sessions per week, and training load per week. The statistical analysis in the groups of volleyball players and gymnasts showed a positive correlation between the competitive experience and regular eating (A1: r = 0.407; *p* = 0.028, A3: r = 0.384; *p* = 0.036). The regularity of eating was much better in girls with higher competitive experience than in the control group. In the group of volleyball players (A1), positive correlations were noted between the number of trainings per week and regular eating (r = 0.475; *p* = 0.009), and between the training load per week and regular eating (r = 0.475; *p* = 0.009). On the other hand, in the group of gymnasts, the correlation between the number of training sessions per week and regular eating proved to be negative (r = −0.470; *p* = 0.009). A statistically significant relationship between the need to reduce body weight and competitive experience was noted in the group of volleyball players (r = −0.589; *p* = 0.001) and gymnasts (r = 0.405; *p* = 0.026). A statistically significant relationship was found between the use of methods for body weight control and the number of training sessions per week in the group of volleyball players (r = −0.370; *p* = 0.048), and between the use of methods for body weight control and the training load per week in group A1 (r = −0.370; *p* = 0.048) and group A3 (r = 0.436; *p* = 0.016), (Table 8).

## 4. Discussion

In the girls training in sports, 27.6% of volleyball players, 33.3% of athletes, and 13.3% gymnasts ate irregularly. The majority of girls in the control and study groups ate regularly, having four main meals during a day. It should be emphasised that the sportswomen groups indicated a greater regularity in eating. No statistically significant differences were observed between the groups, which may result from the number of participants in each group (*p* = 0.078). Furthermore, no restrictions in consumption of individual food groups during a week were observed in the sportswomen or in the control group. Athletes drank significantly more water during the day than women in the control group (*p* < 0.001). Another factor significantly differentiating the groups of girls was vitamin supplementation. The sportswomen took vitamins more frequently (*p* = 0.005). The women who did not regularly take part in sports, drank alcohol significantly more often (*p* = 0.001).

Similar results were obtained by McLester et al., who demonstrated that eating disorders affected 8.6% of female athletes [35]. However, other authors obtained different results.

Torres-McGehee et al. [36] reported a much greater problem with eating in the results of their study. The authors demonstrated that the risk of developing eating disorders affected 76.9%, 72.2%, and 66.7% of young girls training in ballet, beach volleyball, and volleyball, respectively. The results are divergent, partly due to different research tools used, and partly due to the sports disciplines represented by sportswomen. In certain disciplines, the way of eating and peer pressure are factors influencing eating behaviours and body weight of sportswomen.

The results of our study show that studied athletes differed significantly in terms of satisfaction with their body weight (*p* = 0.008). The greatest dissatisfaction was expressed by the volleyball players and the control group, when compared to athletes and gymnasts (A1—83.33%; B—80.00% v. A2—50.00%; A3—53.33%), and those groups also controlled their body weight more frequently. It is worth noting that to control body weight, sportswomen more often reduced their consumption of sweet and salty snacks and carbonated beverages, increased the amount of fruit and vegetables in their diet, and did not eat after 6.00 p.m. In the control group, women restricted their carbohydrate intake and reduced the number of meals eaten. In the group of gymnasts, the need to reduce body weight was the highest when compared to the other groups of sportswomen (A3—56% v. A1—23.33%; A2—30%). In this group, a positive correlation was noted between regularity of eating and the need to reduce body weight (r = 0.049; *p* = 0.013), which may indicate an awareness of the role of eating in the athlete’s diet in this group of sportswomen. In the group of volleyball players, a negative correlation was noted between regular eating of supper and a need to reduce body weight (r = −0.409; *p* = 0.027), an observation confirmed by the described methods for body weight control in this group (not eating after 6.00 p.m.).

Authors of studies focusing on eating disorders in relation to the development of multicomponent FAT syndrome, in girls and young women training sports, note a relationship between incorrect eating behaviours and dissatisfaction with body shape and weight, or the need to reduce body weight. At the same time, they emphasise that the awareness of a relationship between those elements in respect to sports discipline and education in this area may be a tool for the prevention and diagnosis of eating disorders [11,12,13,14,15,16]. In the study by Oliveira et al., changes in eating behaviours and risk factors for development of eating disorders in studied female gymnasts and athletes resulted mainly from their dissatisfaction with their body weight and body image (a positive correlation between dissatisfaction and the occurrence of risky behaviours contributing to the development of eating disorders) [11]. Prnjak confirms that in sportswomen, satisfaction with one’s own body is the greatest factor explaining development of eating disorders [12]. Kong et al., in their study involving 320 sportswomen, indicate a higher level of dissatisfaction with one’s own body and incorrect eating habits in female athletes competing in sports in which a slim body is recommended. Over 60% of sportswomen experienced pressure concerning their body shape from their trainers, to adjust to sports norms for body weight, which poses a risk of developing eating disorders [13]. Suryawati indicates that a negative perception of their body and stress are risk factors for the development of eating disorders in young sportswomen. Eating disorders combined with the negative perception of their body were experienced by 65.8% of studied sportswomen training in different sports disciplines. The authors indicate that when the study subject reports a high level of stress and a negative body image, the probability of developing an eating disorder is 80%. The risk of eating disorders increases tenfold when a sportswoman has a low level of satisfaction with her body, and three times when she experiences stress [14]. This was also demonstrated by Arthur-Cameselle, in studies in elite sportswomen, where risk factors contributing to the development of clinical eating disorders included dissatisfaction with their bodies (83%) and the pressure of sports performance (67%) [10]. Pia Thiemann et al. also note dissatisfaction with one’s own body and the pressure in sport when explaining sportspeople’s susceptibility to incorrect eating behaviours. Sportswomen training in aesthetic sports demonstrated a significantly higher incidence of eating disorders, when compared to sportswomen playing team sports. Dissatisfaction with one’s own body and pressures in sport were significant predictors for incorrect eating behaviours [15]. In Krentz and Warschburger studies conducted on German sportswomen competing in aesthetic sports, a significant influence of social pressure from sports circles was observed. It led to a wish to be slimmer to improve sports capacity, which contributed to dissatisfaction with one’s own appearance and changes in eating behaviours, aimed at reducing body weight [16]. In Reel studies conducted in 280 female gymnasts and 134 female swimmers, authors report the pressure concerning body weight occurring in sports circles subjected by trainers, colleagues from a team, and judges, which in sportswomen may be a cause for the development of unhealthy eating practices to reduce their body weight and change their appearance, to match norms in a given sports discipline. Furthermore, they note that the effect of trainers’ comments concerning body weight and appearance is stronger when a sportswoman is not happy, and feels negative emotions associated with her body weight [18]. In his studies, Thompson also discusses a problem of the pressure concerning body weight and appearance, coming from teammates and coaches, which may significantly contribute to the development of incorrect eating behaviours, and become a reason for complex, multidimensional symptoms of eating disorders [19]. Plateau reports that trainers attributed a drop in sports effectiveness to an increased difficulty of performed exercises, and not to factors associated with eating disorders. They also had a lower tendency for reacting to incorrect eating behaviours when sportsperson’s capacity was good, and they associated changes in eating behaviours in their charges with a wish to increase sports performance [37].

In their studies, Oliveira et al. demonstrated that changes in eating behaviours and risk factors for development of eating disorders were observed in girls training in sports in which body weight control is recommended (gymnastics and athletics) [11]. Roy et al. indicate that the body mass index and care for body shape are the strongest predictors for eating disorders. The greatest number of incorrect eating behaviours were noted in the group of aesthetic sports and in the control group. Furthermore, the frequency of eating products from different food groups was analysed in the study participants. The difference in eating fast food proved to be statistically significant in the control group, while sportspeople training in athletic disciplines (sprint, throw, and jump) were characterised by the highest consumption of sweets [17]. In a study conducted by Villa et al., in a group of elite female gymnasts, it was noted that sportswomen consumed less carbohydrates than the recommended level, which did not supply the required energy, which explains their low body weight and poses a risk to their health and sports capacity [38].

The results of the conducted study and literature report indicate that incorrect eating behaviours occur in girls training in sports. Discrepancies concerning the scale of this problem prompt us to conduct further, more detailed studies in other sports disciplines with larger groups, to verify the obtained results.

### Limitations

In the undertaken study, the study limitations included a retrospective analysis of eating habits as subjectively assessed by girls. This data was collected using a proprietary questionnaire. Furthermore, the study was conducted in sportswomen from the Silesian voivodeship, so its interpretation cannot be generalised.

A problem of eating disorders in girls actively training in sports may represent a starting point for the development of other health disorders, including the Female Athlete Triad. Further studies in a wider population are necessary, and should take into account additional factors and other sports disciplines, to analyse this issue in detail. It is worth noting that this study is a pioneer study in sportswomen from Silesia who represent various sports disciplines, and the presented results are in contrast to studies on similar subjects and provide new knowledge in this area.

## 5. Conclusions

The sportswomen from the Silesian, representing volleyball, athletics, and gymnastics, eat regularly and no worrying eating behaviours were observed in them. Being aware of the risk of eating disorders occurring in sports, and their consequences to health, there is a need to continuously monitor eating habits and diet in women actively training sports. Further studies using a bigger group are necessary to find detailed factors that may increase the likelihood of developing eating disorders.

## Figures and Tables

**Table 1:** Age, height, weight, and BMI of female athletes (A1, A2, A3) and girls from the control group (B).

		M	SD	Me	95%CI		
					Lower Limit	Upper Limit	
Age [years]	A1	16.68	0.80	16.5	16.31	16.93	F (3;118) = 8.67; *p* < 0.001; η² = 0.18
	A2	16.90 ^e^	0.87	17	16.57	17.23	
	A3	16.13 ^a,e^	0.81	16	15.83	16.44	
	B	17.20 ^a^	0.83	17	16.88	17.52	
Body height [cm]	A1	179.17 ^a,b,c^	6.02	178	177.05	181.71	F (3;115) = 33.69; *p* < 0.001; η² = 0.88
	A2	167.27 ^a,^	6.85	168	164.67	169.87	
	A3	163.90 ^b^	6.10	166	161.58	166.22	
	B	165.90 ^c^	6.59	167.5	163.40	168.40	
Body weight [cm]	A1	67.60 ^a,b^	6.57	68.6	64.76	69.64	F (3;115) = 30.69; *p* < 0.001; η² = 0.80
	A2	56.13 ^a,d,g^	6.97	56	53.49	58.78	
	A3	51.21 ^b,g^	5.47	52.1	49.14	53.29	
	B	63.41 ^b,d^	8.59	64.6	60.15	66.68	
BMI	A1	21.06 ^d,f^	1.92	20.94	20.23	21.52	F (3;91.72) = 18.46; *p* < 0.001; η² = 0.32
	A2	20.05 ^b^	2.08	20.00	19.26	20.84	
	A3	19.05 ^a,d^	1.61	18.85	18.44	19.66	
	B	23.04 ^a,b,f^	2.88	22.60	21.93	24.13	

M—mean; SD—standard deviation; Me—median; 95%CI—confidence interval; F—Anova result; degrees of freedom df—provided in brackets; *p*—Anova statistical significance; η^2^—effect size measure; post hoc tests between groups: a,b,c—*p* < 0.001; d—*p* = 0.001; e—*p* = 0.004; f—*p* = 0.007; g—*p* = 0.047.

**Table 2:** Sports activity of groups of the study group.

		M	SD	Me	95%CI		
					Lower Limit	Upper Limit	
Professional internship [years]	A1 ^b^	6.03	1.40	6	5.48	6.59	F (2;78.87) = 9.72; *p* < 0.001; η² = 0.18
	A2 ^a^	6.20	2.04	5.5	5.43	6.97	
	A3 ^a,b^	8.03	2.18	8	7.20	8.86	
Duration of 1 training [h]	A1 ^d^	2.00	0.00	2	2.00	2.00	F (2;86) = 11.05; *p* < 0.001; η² = 0.20
	A2 ^c^	2.07	0.44	2	1.90	2.23	
	A3 ^c,d^	2.60	0.80	2	2.30	2.90	
Total training time per week [h]	A1	14.20	5.38	16	11.93	16.07	F (2;78.13) = 2.50; *p* = 0.088; η² = 0.05
	A2	12.07	3.89	12	10.59	13.54	
	A3	15.03	5.91	14	12.79	17.28	

M—mean; SD—standard deviation; Me—median; Min-Max—minimum and maximum values; F—ANOVA result; degrees of freedom df—provided in brackets; η^2^—effect size measure. a: *p* = 0.005, b: *p* < 0.001, c: *p* = 0.009, d: *p* = 0.001.

**Table 3:** Frequency of consuming specific foods during a week and volume of fluids drank a day in groups A1, A2, A3 and B.

	A1		A2		A3		B		
	M	SD	M	SD	M	SD	M	SD	
Dairy	5.41	2.228	6.17	1.487	5.70	1.915	5.43	1.977	F (3;106.47) = 0.99; *p* = 0.399; η² = 0.02
Fish	1.41 ^d^	0.946	1.23	0.568	0.93 ^d^	0.583	1.07	0.521	F (3;85.97) = 2.82; *p* = 0.046; η² = 0.06
Meat and cold cuts	4.72	2.266	4.50	1.852	4.97	2.042	3.80	2.325	F (3;111.29) = 1.669; *p* = 0.178; η² = 0.04
Flour products, pasta, groats and rice	5.21	1.953	5.23	2.096	4.57 ^e^	2.648	6.10 ^e^	1.539	F (3;100.73) = 2.70; *p* = 0.049; η² = 0.06
Fruit and vegetables	6.41	1.053	5.83	1.683	5.80	1.750	5.90	1.989	F (3;101.63) = 0.88; *p* = 0.451; η² = 0.02
Salty and sweet snacks	4.31	2.189	3.77	2.285	3.40	2.298	2.87	2.047	F (3;114.09) = 2.24; *p* = 0.087; η² = 0.05
Fluids and water	2.43 ^c^	0.417	2.48 ^a^	0.737	2.45 ^b^	0.356	1.92 ^a,b,c^	0.657	F (3;89.37) = 6.81; *p* < 0.001; η² = 0.15

M—mean; SD—standard deviation; F—Anova test result, degrees of freedom df—provided in brackets; *p*—statistical significance; η2—effect size measure. a: *p* = 0.016, b: *p* = 0.002, c: *p* = 0.004, d: *p* = 0.042, e: *p* = 0.05.

**Table 4:** Characteristics of regularity of vitamins and dietary supplements taking in groups A1, A2, A3, and B.

		A1		A2		A3		B		
		n	%	n	%	n	%	n	%	*p*
Vitamins	Yes	19	63.33	18	60.00	12	40.00	7	23.33	χ^2^(3) = 12.01 *p* = 0.005 V = 0.38
	Not	11	36.67	12	40.00	18	60.00	23	76.67	
Dietary supplements	Yes	8	26.67	7	23.33	4	13.33	8	26.70	χ^2^(3) = 2.17 *p* = 0.564 V = 0.13
	Not	22	73.33	23	76.67	26	86.67	23	73.30	

χ^2^—test result; degrees of freedom—df provided in brackets; *p*—statistical significance; V—effect size.

**Table 5:** Use of stimulants in the study groups and in the control group.

		A1		A2		A3		B		
		n	%	n	%	n	%	n	%	*p*
Cigarettes	Yes	3	10.00	2	6.67	0	0.00	2	6.67	Exact Fisher Test *p* = 3.165
	Not	27	90.00	28	93.33	30	100.00	28	93.33	
Alcohol	At all	12	40.00	4	13.33	15	50.00	5	16.67	χ^2^(6) = 23.28 *p* = 0.001 V = 0.31
	Occasionally	12	40.00	22	73.33	10	33.33	12	40.00	
	Regularly	6	20.00	4	13.33	5	16.67	13	43.33	

χ^2^—test result; degrees of freedom—df provided in brackets; *p*—statistical significance; V—effect size.

**Table 6:** Satisfaction with body weight and use of methods for body weight control in the studied and in the control groups.

		A1		A2		A3		B		
		n	%	n	%	n	%	n	%	*p*
Satisfaction with body weight	Yes	5	16.67	15	50.00	14	46.67	6	20.00	χ^2^(3) = 11.88 *p* = 0.008 V = 0.32
	Not	25	83.33	15	50.00	16	53.33	24	80.00	
Need to lose weight	Yes	7	23.33	9	30.00	17	56.70	-	-	χ^2^(2) = 12.62 *p* = 0.002 V = 0.37
	Not	23	76.67	21	70.00	13	43.33	-	-	
Use of weight control methods	Yes	16	53.33	9	30.00	6	20.00	16	53.33	χ^2^(3) = 10.16 *p* = 0.017 V = 0.29
	Not	14	46.67	21	70.00	24	80.00	14	46.67	

χ^2^—test result; degrees of freedom—df provided in brackets; *p*—statistical significance; V—effect size.

**Table 7:** Eating habits and satisfaction with body weight, a need to reduce it, and the use of methods for body weight control.

		Satisfaction with Body Weight		Need to Reduce Weight		Use of Methods for Body Weight Control	
		r Pearson	*p*	r Pearson	*p*	r Pearson	*p*
Regular nutrition	A1	0.282	0.139	−0.348	0.064	−0.287	0.130
	A2	−0.141	0.456	0.154	0.416	0.154	0.416
	A3	−0.419	0.021	0.449	0.013	−0.049	0.797
Regular I breakfast	A1	0.139	0.472	−0.240	0.210	0.025	0.987
	A2	0.000	1.000	−0.036	0.849	−0.036	0.849
	A3	−0.223	0.237	0.251	0.182	−0.049	0.797
Regular II breakfast	A1	0.139	0.472	0.218	0.255	0.025	0.897
	A2	−0.333	0.072	−0.036	0.849	−0.036	0.849
	A3	−0.200	0.288	0.235	0.210	−0.167	0.379
Regular lunch	A1	0.208	0.278	−0.257	0.178	−0.076	0.697
	A2	0.186	0.326	0.122	0.522	0.122	0.522
	A3	−0.223	0.237	0.053	0.782	0.196	0.299
Regular dinner	A1	0.331	0.079	−0.409	0.027	−0.265	0.164
	A2	−0.069	0.716	0.045	0.812	0.045	0.812
	A3	−0.191	0.311	0.233	0.215	0.302	0.105

Pearson’s r—Paerson’s r test; *p*—statistical significance.

**Table 8:** Eating habits, satisfaction with body weight, a need to reduce it, and the use of methods for body weight control versus competitive experience, the number of training sessions per week and, training load per week.

		Competitive Experience [Years]		Number of Trainings Per Week		Training Load Per Week [h]	
		r Pearson	*p*	r Pearson	*p*	r Pearson	*p*
Regular nutrition	A1	0.407	0.028	0.475	0.009	0.475	0.009
	A2	0.163	0.390	0.043	0.822	−0.077	0.688
	A3	0.384	0.036	−0.470	0.009	−0.104	0.585
Satisfaction with body weight	A1	0.084	0.665	0.039	0.839	0.839	0.839
	A2	0.193	0.307	−0.028	0.882	0060	0.752
	A3	−0.086	0.652	0.064	0.736	−0.035	0.853
Need to reduce weight	A1	−0.589	0.001	0.218	0.255	0.218	0.255
	A2	0.348	0.060	0.044	0.817	0.166	0.380
	A3	0.405	0.026	−0.008	0.966	0.229	0.223
Use of methods for body weight control	A1	−0.085	0.662	−0.370	0.048	−0.370	0.048
	A2	0.348	0.060	0.044	0.817	0.166	0.380
	A3	0.171	0.368	0.330	0.075	0.436	0.016

r Pearson—Paerson’s r test; *p*—statistical significance.

## References

[1] Langley-Evans S.C., Jarosz M. (2014). Żywienie: Wpływ na Zdrowie Człowieka.

[2] Llorente-Cantarero F., Palomino-Fernández L., Gil-Campos M. (2018). Nutrition for the Young Athlete. J. Child. Sci..

[3] Thomas D.T., Erdman K.A., Burke L.M. (2016). American College of Sports Medicine joint position statement. Nutrition and athletic performance. Med. Sci. Sport. Exerc..

[4] Morris J. (2011). ABC of Eating Disorders.

[5] Birmingham C.L., Treasure J. (2010). Medical Management of Eating Disorders.

[6] Bratland-Sanda S., Sundgot-Borgen J. (2013). Eating disorders in athletes: Overview of prevalence, risk factors and recommendations for prevention and treatment. Eur. J. Sport Sci..

[7] Reinking M.F., Alexander L.E. (2005). Prevalence of disordered-eating behaviours in undergraduate female collegiate athletes and nonathletes. J. Athl. Train..

[8] Keski-Rahkonen A., Hoek H.W., Susser E.S., Linna M.S., Sihvola E., Raevuori A., Bulik C.M., Kaprio J., Rissanen A. (2007). Epidemiology and course of anorexia nervosa in the community. Am. J. Psychiatry.

[9] Smink F.R., van Hoeken D., Hoek H.W. (2012). Epidemiology of eating disorders: Incidence, prevalence, and mortality rates. Curr. Psychiatry Rep..

[10] Arthur-Cameselle J., Sossin K., Quatromoni P. (2017). A qualitative analysis of factors related to eating disorder onset in female collegiate athletes and non-athletes. Eat Disord..

[11] Oliveira G.L., Oliveira T.A.P., Gonçalves P.S.P., Valentim-Silva J.R., Fernandes P.R., Fernandes Filho J. (2017). Body image and eating disorders in female athletes of different sports. J. Exerc. Physiol..

[12] Prnjak K., Jukic I., Tufano J.J. (2019). Perfectionism, body satisfaction and dieting in athletes: The role of gender and sport type. Sports.

[13] Kong P., Harris L.M. (2015). The sporting body: Body image and eating disorder symptomatology among female athletes from leanness focused and nonleanness focused sports. J. Psychol..

[14] Suryawati D.F.F., Purwanti R., Tsani A.F.A., Widyastuti N. (2020). Risk factors of eating disorders in young female athletes. Food Res..

[15] Thiemann P., Legenbauer T., Vocks S., Platen P., Auyeung B., Herpertz S. (2015). Eating disorders and their putative risk factors among female german professional athletes. Eur. Eat. Disorders Rev..

[16] Krentz E.M., Warschburger P. (2011). Sports-related correlates of disordered eating in aesthetic sports. Psychol. Sport Exerc..

[17] Roy M., Chatterjee S., Dey S.K. (2019). Interrelationship of stress, body image, negative mood state and susceptibility to abnormal eating disorders among game specific female athlete. Eur. J. Phys. Educ. Sport.

[18] Reel J.J., Petrie T.A., SooHoo S., Anderson C.M. (2013). Weight pressures in sport: Examining the factor structure and incremental validity of the weight pressures in sport e females. Eat. Behav..

[19] Thompson R.A., Sherman R. (2014). Reflections on athletes and eating disorders. Psychol. Sport Exerc..

[20] Sundgot-Borgen J. (1993). Prevalence of eating disorders in elite female athletes. Int. J. Sport Nutr..

[21] Sundgot-Borgen J., Torstveit M.K. (2004). Prevalence of eating disorders in elite athletes is higher than in the general population. Clin. J. Sport Med..

[22] Sundgot-Borgen J., Torstveit M.K. (2010). Aspects of disordered eating continuum in elite high intensity sports. Scand. J. Med. Sci. Sport..

[23] Nattiv A., Loucks A.B., Manore M.M., Sanborn C.F., Sundgot-Borgen J., Warren M.P. (2007). American College of Sports Medicine position stand. The female athlete triad. Med. Sci. Sport. Exerc..

[24] De Souza M.J., Nattiv A., Joy E., Misra M., Williams N.I., Mallinson R.J., Matheson G. (2014). 2014 Female Athlete Triad Coalition Consensus Statement on Treatment and Return to Play of the Female Athlete Triad: 1st International Conference held in San Francisco, California, May 2012 and 2nd International Conference held in Indianapolis, Indiana, May 2013. Br. J. Sport. Med..

[25] Loveless M., Hewitt G. (2017). Committee Opinion No.702: Female Athlete Triad. Obstet. Gynecol..

[26] Mehta J., Thompson B., Kling J.M. (2018). The female athlete triad: It takes a team. Clev. Clin. J. Med..

[27] Ackerman K.E., Holtzman B., Cooper K.M., Flynn E.F., Bruinvels G., Tenforde A.S., Popp K.L., Simpkin A.J. (2019). Low energy availability surrogates correlate with health and performance consequences of Relative Energy Deficiency in Sport. Br. J. Sport. Med..

[28] Skrzypulec V., Lindert O., Morawiec M., Nowosielski K., Drosdzol A., Klimanek M. (2005). Zaburzenia miesiączkowania u sportsmenek. Ginekol. Prakt..

[29] Wodarska M., Witkoś J., Drosdzol-Cop A., Dąbrowska J., Dąbrowska-Galas M., Hartman M., Plinta R., Skrzypulec-Plinta V. (2013). Menstrual cycle disorders in female volleyball players. J. Obstet. Gynaecol..

[30] Czajkowska M., Drosdzol-Cop A., Gałązka I., Naworska B., Skrzypulec-Plinta V. (2015). Menstrual cycle and the prevalence of premenstrual syndrome/premenstrual dysphoric disorder in adolescent athletes. J. Pediatr. Adolesc. Gynecol..

[31] Czajkowska M., Drosdzol-Cop A., Naworska A., Galazka I., Gogola C., Rutkowska M., Skrzypulec-Plinta V. (2020). The impact of competitive sports on menstrual cycle and menstrual disorders, including premenstrual syndrome, premenstrual dysphoric disorder and hormonal imbalances. Ginekol. Pol..

[32] Czajkowska M., Plinta R., Rutkowska M., Brzęk A., Skrzypulec-Plinta V., Drosdzol-Cop A. (2019). Menstrual cycle disorders in professional female rhythmic gymnasts. Int. J. Environ. Res. Public Health.

[33] Coelho G.M., Gomes A.I., Ribeiro B.G. (2014). Soares Ede, A. Prevention of eating disorders in female athletes. Open Access J. Sport. Med..

[34] George D., Mallery P. (2010). SPSS for Windows Step by Step: A Simple Guide and Reference 17.0 Update.

[35] McLester C.N., Hardin R., Hoppe S. (2014). Susceptibility to eating disorders among collegiate female student–athletes. J. Athl. Train..

[36] Torres-McGehee T.M., Emerson D.M., Pritchett K., Moore E.M., Smith A.B., Uriegas N.A. (2021). Energy availability with or without eating disorder risk in collegiate female athletes and performing artists. J. Athl. Train..

[37] Plateau C.R., McDermott H.J., Arcelus J., Meyer C. (2014). Identifying and preventing disordered eating among athletes. Psychol. Sport Exerc..

[38] Villa M., Villa-Vicente J.G., Seco-Calvo J., Mielgo-Ayuso J., Collado P.S. (2021). Body composition, dietary intake and the risk of low energy availability in elite-level competitive rhythmic gymnasts. Nutrients.

